# Supplementary material for: Microwave-Assisted Valorization of Tomato Pomace for Pectin Recovery: Improving Yields and Environmental Footprint
Source: Foods. 2025 Apr 26;14(9):1516. doi: 10.3390/foods14091516 (PMC12071326; doi:10.3390/foods14091516)
Supplement: Supplementary file 1 [file foods-14-01516-s001.zip › Figure S1.pdf]

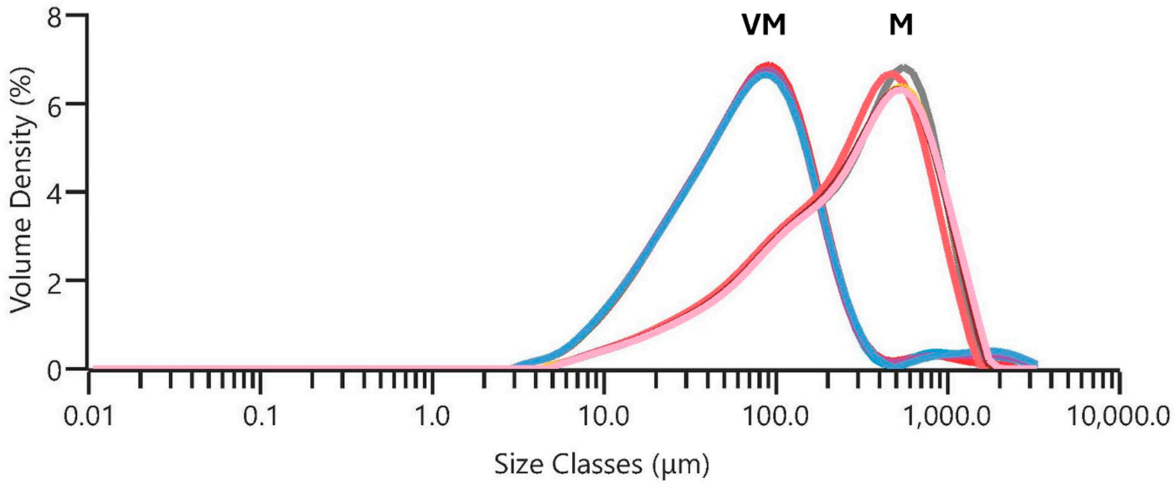

Sample	Dv 10 (µm)	Dv 50 (µm)	Dv 90 (µm)	D [4,3] (µm)	D [3,2] (µm)
VM	16.6 ± 0.7	66.8 ± 0.4	191.0 ± 4.8	122.8 ± 12.8	38.1 ± 0.2
M	43.6 ± 1.6	303.0 ± 13.8	849.0 ± 54.2	382.2 ± 20.8	102.3 ± 3.4

Figure S1. Impact of pretreatment in vibrational micromill on the particle size distribution of TPW.
