# Supplementary material for: Microwave-Assisted Valorization of Tomato Pomace for Pectin Recovery: Improving Yields and Environmental Footprint
Source: Foods. 2025 Apr 26;14(9):1516. doi: 10.3390/foods14091516 (PMC12071326; doi:10.3390/foods14091516)
Supplement: Supplementary file 1 [file foods-14-01516-s001.zip › Table S1 -novo.pdf]

Table S1. ANOVA and equation coefficients (coded factors) of the quadratic model for the yield

	F-value	p-value	Coefficient Estimate
**Model**	2.98	0.052	Intercept 6.65
**A-Time**	3.88	0.0772	0.9789
**B-Power**	0.809	0.3895	-0.4276
**C-pH**	0.3984	0.5421	0.3
**AB**	8.74	0.0144	-1.84
**AC**	0.0132	0.9109	-0.0713
**BC**	2.15	0.1731	0.9113
**A^2**	11.35	0.0071	-1.76
**B^2**	0.9615	0.3499	-0.4524
**C^2**	0.2117	0.6553	0.2123
