# Supplementary material for: Microwave-Assisted Valorization of Tomato Pomace for Pectin Recovery: Improving Yields and Environmental Footprint
Source: Foods. 2025 Apr 26;14(9):1516. doi: 10.3390/foods14091516 (PMC12071326; doi:10.3390/foods14091516)
Supplement: Supplementary file 1 [file foods-14-01516-s001.zip › Table S2 -novo.pdf]

Table S2. ANOVA and equation coefficients (coded factors) of the linear model for the degree of esterification

	F-value	p-value	Coefficient Estimate
**Model**	4.83	0.014	Intercept 36.08
**A-Time**	1.55	0.2309	4.29
**B-Power**	0.2459	0.6267	-1.65
**C-pH**	12.7	0.0026	11.88
